# An Environmental Scan of Existing Canadian Childcare Resources Targeting Improvements in Health Behaviours

**DOI:** 10.1007/s10643-021-01266-2

**Published:** 2021-09-28

**Authors:** Valerie Carson, Madison Predy, Stephen Hunter, Kate Storey, Anna P. Farmer, Jessie-Lee McIsaac, Jenn Flynn

**Affiliations:** 1 Faculty of Kinesiology, Sport and Recreation, University of Alberta, Edmonton, AB Canada; 2 School of Public Health, University of Alberta, Edmonton, AB Canada; 3 Faculty of Agricultural, Life and Environmental Sciences, University of Alberta, Edmonton, AB Canada; 4 Department of Child and Youth Study, Faculty of Education, Mount Saint Vincent University, Halifax, NS Canada; 5 APPLE Schools, Edmonton, AB Canada

**Keywords:** Child care, Environmental scan, Nutrition, Physical activity, Sedentary behaviour, Sleep

## Abstract

The objective was to conduct an environmental scan of existing Canadian childcare resources targeting nutrition, physical activity, sedentary behaviour, and/or sleep. A comprehensive search plan was conducted that involved four search strategies: (1) grey literature databases, (2) customized Google search engines, (3) targeted websites, and (4) consultation with content experts. A resource (i.e., information, materials) must have been created by government or an organization/agency within Canada, available in English, intended for childcare educators or directors working with children ≤ 5 years of age, and focused on targeting improvements in nutrition, physical activity, sedentary behaviour, and/or sleep. The quality of each included resource was assessed using a modified version of the Authority, Accuracy, Coverage, Objectivity, Date, Significance checklist. A total of 192 eligible resources were included. Most resources targeted only nutrition (n = 101) or physical activity (n = 60), and few resources targeted only sedentary behaviour (n = 2) or sleep (n = 1). The remaining 28 resources targeted more than one health behaviour. The 4 most common topics of resources were menu/meal planning (n = 55), healthy nutrition practices/environment (n = 37), physical activities/games (n = 33), and nutrition/food literacy (n = 20). Only 52 included resources cited evidence. One-third of the included resources (n = 64) were rated as high quality, including 55 that received a point for the significance criterion. Therefore, numerous high quality Canadian childcare resources exist for nutrition and physical activity. Future resource development is needed for sedentary behaviour and sleep. Findings can assist future intervention work and the database of resources can be utilized by relevant stakeholders to support other childcare initiatives.

**Supplementary Information:**

The online version contains supplementary material available at 10.1007/s10643-021-01266-2.

## Introduction

The first 5 years of life, also known as early childhood, is a period where healthy development in physical, cognitive, and social-emotional domains is paramount for future health (Thompson, 2001). The importance of providing guidance and support for a nutritious diet, regular physical activity (including active outdoor play), minimal sedentary behaviour (in particular screen time), and adequate sleep during early childhood has been recognized by the World Health Organization (WHO) Commission on Ending Childhood Obesity (World Health Organization, 2016). This key area of action outlined by the WHO is informed by the growing evidence on the role these health behaviours play in strengthening children’s healthy development (Alles et al., 2014; Carson et al., 2017; Chaput et al., 2017b; Okely et al., 2018; Poitras et al., 2017). Additionally, these healthy behavioural patterns established at a young age can continue beyond early childhood (Alles et al., 2014; Jones et al., 2013; Mikkila et al., 2005), where there are additional links with health benefits (Aune et al., 2017; Carson et al., 2016; Chaput et al., 2016; Poitras et al., 2016; Warburton et al., 2010). Unfortunately, many young children are not meeting health behaviour guidelines, including the WHO Guidelines on Physical Activity, Sedentary Behavior, and Sleep for Children Less Than 5 Years of Age (Juana & Fiona, 2020). For example, in Canada, most young children (87–95%) do not meet these international or national guidelines on physical activity, sedentary behaviour, and sleep (Carson et al., 2019; Chaput et al., 2017a; Lee et al., 2017). Furthermore, the majority of children (61–73%) do not usually meet the recommended intake of fruits and vegetables (Polsky & Garriguet, 2020). Thus, early promotion of healthy behavioural patterns may be an important strategy to foster a cost-effective trajectory of healthy development (Cunha et al., 2006).

Childcare centres are ideal settings to support healthy development in many young children. For example, approximately 60% of Canadian children aged 5 years and younger (~ 1.4 million children) attend some form of non-parental childcare (Findlay, 2019). Similar percentages are observed internationally, with 34% of 1-year-olds, 46% of 2-year-olds, and 88% of 3–5-year-olds attending early childhood education programs in Organization for Economic Cooperation and Development (OECD) countries (OECD, 2020). Additionally, compared to other settings, such as schools, childcare settings have unique opportunities to promote healthy patterns of nutrition, physical activity, sedentary behaviour, and sleep. For example, unlike schools, many childcare centres provide food for children (Larson et al., 2011) and often have nap or rest times, given the sleep needs of young children (Galland et al., 2012). Also, childcare settings typically have less structured learning compared to school settings, allowing more opportunities for active outdoor play (Dietze & Kashin, 2019). However, childcare directors and educators may not have sufficient training, knowledge, and skills regarding these health behaviours. For instance, previous research in Canada has shown childhood educators receive minimal to no formal training in physical activity (Bruijns et al., 2019; Martyniuk & Tucker, 2014). Studies in the United States have also shown that childcare directors and educators have low knowledge related to nutrition (Nahikian-Nelms, 1997; Rida et al., 2018; Sharma et al., 2013).

A promising strategy to promote healthy development and reduce the risk of childhood obesity is to build capacity and provide appropriate training and professional development to childcare directors and educators regarding promoting healthy behavioural patterns (Dunn-Carver et al., 2013; Jones et al., 2011; Robinson et al., 2012; Waters et al., 2011; World Health Organization, 2016). Government, universities, and health professional organizations have been identified as key institutions in supporting this strategy (World Health Organization, 2016). Taking advantage of existing health behaviour resources developed by multiple institutions for the unique needs of childcare settings, could help support this work (Fees et al., 2009; Jones & Zidenberg-Cherr, 2015; Tucker et al., 2011). For instance, previous research has found that childcare providers identified resources as integral to making changes to support nutrition and physical activity (Elias et al., 2020). However, it has been found that educators lack awareness of health behaviour resources (Jones & Zidenberg-Cherr, 2015) and have expressed a need for additional free, higher quality resources (Fees et al., 2009; Tucker et al., 2011).

To our knowledge, a Canadian database of childcare nutrition, physical activity, sedentary behaviour, and sleep resources does not exist, and the quality of existing resources has not been previously assessed. Therefore, we conducted an environmental scan of existing childcare health behaviour resources in Canada. Environmental scans are useful in strategic planning and project development (Charlton et al., 2019), and they aim to “understand context; collect information; and identify resources, links, and gaps” (Wilburn et al., 2016, p. 1). The specific objectives of this environmental scan were to: identify existing Canadian childcare resources targeting improvements in nutrition, physical activity, sedentary behaviour, and/or sleep; assess the quality of existing resources; create a database of resources for use in future intervention research and initiatives by stakeholders in the childcare community; and identify resource gaps to inform future resource development in this area.

## Methods

### Eligibility Criteria

Childcare resources were broadly defined as information and materials targeted at childcare educators (e.g., ideas for physically active games or encouraging healthy eating habits) and/or directors (e.g., policy development, menu planning) to improve health behaviours in children ≤ 5 years. Specifically, to be eligible for this environmental scan, a resource must have been: (1) created by the government or an organization/agency within Canada, (2) available in English, (3) intended for childcare educators and/or directors working with children ≤ 5 years of age, and (4) targeting improvements in at least one of the four health behaviours of interest, including nutrition, physical activity, sedentary behaviour, and sleep. Nutrition was defined as, the study of food, how food nourishes our bodies, as well as the factors that influence our food intake (Thompson et al., 2010). Physical activity was defined as, “any bodily movement generated by skeletal muscles that causes energy expenditure above resting levels” (Caspersen et al., 1985, p. 126). Sedentary behaviour was defined as, “any waking behavior characterized by an energy expenditure ≤1.5 metabolic equivalents (METs), while in a sitting, reclining or lying posture” (Tremblay et al., 2017a). Sleep was defined as, “a naturally recurring state of body and mind characterized by altered consciousness, relatively inhibited sensory activity, inhibition of nearly all voluntary muscles and reduced interactions with surroundings” (Chaput et al., 2017c, p. 8).

Information on common procedures (e.g., safe handling of food, safe sleep practices) or fact sheets related to a health behaviour (e.g., general facts about physical activity) were not included as these types of resources either provided only basic safety information or did not provide guidance on how to improve the selected health behaviour. All modes of resources were eligible, including traditional print modes (e.g., information sheet, manual) and virtual modes (e.g., webpage, webinar). However, information or materials that were not publicly available (i.e., fee required to access) or could not be accessed (e.g., broken web link, not released yet, in-person) were excluded because we could not assess inclusion criteria or complete a quality assessment. If a resource had several drafts/versions, the newest or most up to date version of the resource was retained and the previous drafts/versions were excluded, including resources that solely focused on the old Canada’s Food Guide (Health Canada, 2007).

### Information Sources and Search Strategy

The environmental scan search plan followed a template for applying systematic review search methods to the grey literature (Godin et al., 2015) and was reviewed by a librarian with expertise in systematic literature searches. Grey literature is defined as “that which is produced on all levels of government, academics, businesses and industry in print and electronic formats, but which is not controlled by commercial publishers, i.e., where publishing is not the primary activity of the producing body” (Farace & Frantzen, 2004). The plan included four different grey literature search strategies: (1) grey literature databases, (2) customized Google search engines, (3) targeted websites, and (4) consultation with content experts. These searches were carried out between September, 2019 and March, 2020 by two researchers.

For the first search strategy, six grey literature databases that were relevant to our environmental scan topic were searched by one researcher between September and October, 2019. The databases searched included Canadian Agency for Drugs and Technologies Health (CADTH), Canadian Best Practices Portal, Health Systems Evidence, Canadian Health & Human Resources (CHHRRN/CIHI) Library, Public Health Grey Literature, and Turning Research into Practice (TRIP). See supplementary file 1 for the specific search strategies.

For the second search strategy, three customized Google search engines were searched between September and November, 2019 by one researcher. The customized Google search engines included Canadian Public Health Associations, Ontario Public Health Unit Websites, and Canadian Public Health Information. Therefore, these searches targeted resources that were created by public health agencies. Due to some operational issues with the Canadian Public Health Information, the customized Google search engine had to be re-created by a member of the research team, and only 10 websites could be searched at a time. See supplementary file 2 for the specific search strategies. The first 10 pages or 100 results were screened for each search.

For the third search strategy, relevant websites of government and organizations/agencies that were not included in the customized Google search engines outlined in the second search strategy were identified by the research team. Advanced Google searches were then conducted between November, 2019 and March, 2020 by two researchers, who each did approximately half. The searches were used to identify websites of relevant government and organizations/agencies that had not already been identified by the research team or the second search strategy. The same search strategies that were used for the customized Google search engines were applied (See supplementary file 2). Additionally, the region of Canada was selected in the advanced search. The first 10 pages or 100 results were screened for each search. See supplementary file 3 for a list of websites searched.

For the fourth search strategy, one researcher contacted content experts via email between October and November, 2019. Content experts were given a brief introduction to the study and an outline of the eligibility criteria for resources. A total of 27 individuals were contacted that represented relevant Canadian researchers and key stakeholders in the childcare community, primarily in Alberta, Canada. The project team selected Canadian researchers who had previously published work on at least one of the health behaviours of interest within childcare settings. The research team selected stakeholders who represented key areas of the childcare sector (e.g., licensing, accreditation, curriculum). For those who did not respond to the original email, one follow-up email was sent 2 weeks later.

For all four search strategies, initial screening was conducted by the researcher who did the search and involved reviewing titles and where applicable, short text beneath the title. For the third and fourth search strategy, if the record was a website, the website was searched using the website’s search bar and the same combination of search terms as the original search. If the website did not have a search bar the website was searched manually. Potentially relevant records were bookmarked (or for the fourth search strategy some records were saved to the computer) and title, organization, and URL (if applicable) were recorded in Excel. Duplicates within each search strategy were not included. After the four search strategies were completed, all potentially relevant records were compiled in Excel and duplicates were removed. Next, between April and June, 2020, one researcher screened abstracts, executive summaries, and/or tables of contents for each record. Those records that did not meet the eligibility criteria were excluded and the reasons for exclusion were noted. Initial screening was completed by one researcher because of budget and timeline restraints. Then the full text of the remaining records were screened by two independent researchers. To be included or excluded, agreement was required and any disagreements were resolved by a third researcher. Reasons for exclusion were noted where applicable.

### Data Extraction

Characteristics of each included resource were extracted and recorded in Excel by one researcher between June and September, 2020. This information included the health behaviour(s) targeted (i.e., nutrition, physical activity, sedentary behaviour, sleep), source, URL, year published, inclusion of goal/objective, intended audience (i.e., educators and/or directors), mode (e.g., information sheet, webinar), inclusion of cited evidence, and health behaviour topic(s) (e.g., healthy practices/environment, menu/meal planning).

### Quality Assessment

To determine the resources that may be most helpful in supporting future intervention research and other childcare initiatives, the quality of each included resource was assessed. Two independent researchers assessed resource quality between June and August 2020 using a modified version of the Authority, Accuracy, Coverage, Objectivity, Date, Significance (AACODS) checklist (Tyndall, 2010). This tool has specifically been developed for quality assessment of grey literature sources (Tyndall, 2010). Briefly, the tool includes six assessment criteria, and each criterion had a question or series of questions. Some of the questions were modified by the research team so they were applicable for this environmental scan. Specifically, for the Authority criterion we used two questions (reputable, authority in the field), for the Accuracy criterion we used three questions (aim/brief, references, representative of field), for the Coverage criterion we used one question (limits stated), for the Objective criterion we used two questions (clear standpoint, balanced), for the Date criterion we used two questions (clear date, recent date), and for the Significance criterion we used three questions (meaningful, unique, impactful). Each question had three response options, including yes, no, and unsure. If the majority of questions were scored yes, then the criterion was given a point. For the final score, the points were added up across criteria, and a score of ≤ 2 was categorized as low-quality, 3–4 as moderate-quality, and ≥ 5 as high-quality. Discrepancies in criterion and final scores were resolved with discussion between the two researchers. Inter-rater reliability prior to discussions was determined by calculating Cohen’s Kappa coefficient and were defined as: poor/slight (К = 0.00–0.20), fair (К = 0.21–0.40), moderate (К = 0.41–0.60), substantial (К = 0.61–0.80), and almost perfect (К = 0.81–1.00) (Landis & Koch, 1977). The final agreed-upon scores and quality rating were added to the Excel file that included the characteristics of resources.

### Synthesis of Results

Frequencies and/or proportions were calculated to describe the characteristics of resources, including health behaviour(s) targeted, types of sources, inclusion of a goal/objective, intended audience, types of modes, inclusion of cited evidence, and health behaviour topics. For types of sources and modes, frequencies were calculated across health behaviours and for each health behaviour. Frequencies were also calculated to describe the quality of resources across health behaviours and for each health behaviour. For some high-quality resources, including those that received a point for the significance criterion and those that received a point for all criteria, frequencies were calculated for each health behaviour, mode, and topic. When additional frequencies were calculated separately for each health behaviour, resources that targeted more than one health behaviour were combined into a combination category. Finally, the range in publication years, when available, was also calculated.

## Results

### Number of Resources

**Fig. 1:**
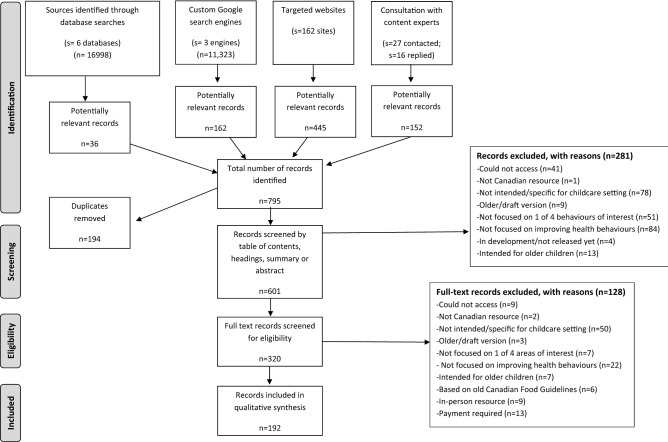
Flow diagram for the identification, screening, eligibility, and inclusion of resources

Across the four grey literature search strategies, a total of 795 potentially relevant records were identified (see Fig. 1). After 194 duplicates were removed, the abstracts, summaries, and table of contents were screened for 601 potentially relevant records, and 320 potentially relevant records were kept for full-text screening. After full-text screening, 192 records met the inclusion criteria of a childcare resource and were included in the study. Reasons for excluding records are outlined in detail in Fig. 1.

### Characteristics of Resources

**Fig. 2:**
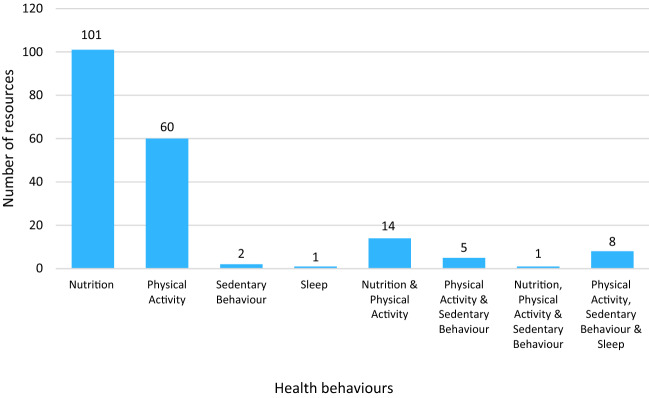
The health behaviour or behaviours targeted by included resources

A database of included resources, characteristics of the resources, and quality assessment scores can be found in supplementary file 4. The health behaviour or behaviours targeted across the 192 resources is displayed in Fig. 2. Of the resources that targeted a single behaviour, the majority were focused on nutrition (n = 101) or physical activity (n = 60), whereas few resources focused on sedentary behaviour (n = 2) or sleep (n = 1). Of the remaining 28 resources that targeted multiple behaviours, half targeted both nutrition and physical activity (n = 14). No resource targeted all 4 behaviours.

Of the 192 included resources, 133 (69%) had a clearly stated goal or objective but only 52 (27%) resources cited evidence. Of the 72 resources that provided a date of publication, it ranged between 2005 and 2019. Many resources were intended for directors (n = 58), educators (n = 76), or both educators and directors (n = 58). Resources were released by various types of sources (see Table 1), with the most common types of sources across health behaviours being public health authority (n = 56), provincial/territorial government (n = 53), and behaviour specific group (n = 52). Government and public health authorities accounted for 81% of nutrition resources, compared to only 37% of physical activity resources, and only one government resource was found that included multiple behaviours. There were 10 different modes of resources across behaviours (see Table 1), with the most common being information sheet/poster (n = 64), manual (n = 39), and activity/recipe booklet/database (n = 37). Information sheet/posters was the most common mode for nutrition, accounting for 39% of nutrition resources, whereas activity booklet/database was the most common mode for physical activity, accounting for 31% of physical activity resources.

**Table 1:** Types of sources and modes of included resources stratified by health behaviour

Source	Nutrition	Physical activity	Sedentary behaviour	Sleep	Combination^a^	Total
	# of resources	# of resources	# of resources	# of resources	# of resources	# of resources
Federal Government	4	0	0	0	0	4
Provincial or Territorial Government	39	12	0	1	1	53
Public Health Authority	39	10	1	0	6	56
Behaviour Specific Group	9	25	1	0	17	52
Childcare Specific Group	5	9	0	0	4	18
University/ Research Group	5	4	0	0	0	9

Mode	# of resources	# of resources	# of resources	# of resources	# of resources	# of resources^b^
Activity booklet/database	7	19	0	0	4	30
Activity planner	0	2	0	0	0	2
Assessment tool	4	1	0	0	3	8
Info sheet/poster	40	18	1	1	4	64
Manual	24	8	1	0	6	39
Menu template/sample menu	8	0	0	0	1	9
Online course/module	3	2	0	0	4	9
Recipe booklet/database	8	1	0	0	0	9
Video	0	4	0	0	4	8
Webinar	4	2	0	0	0	6
Webpage	4	4	0	0	2	10

^a^The combination category includes resources that targeted more than one health behavior

^b^The total number of resources for mode does not equal the number of included resources because two resources were classified as two modes

A summary of topics covered by resources for each health behaviour can be found in Table 2. The most common resource topics for nutrition were menu/meal planning (n = 55), healthy practices/environment (e.g., educator role modelling, respecting children’s satiety cues; n = 37), and nutrition/food literacy (e.g., teaching children about nutrition/food; n = 20). The most common resource topics for physical activity were games/activities (n = 34) and physical literacy (n = 13) defined as “the motivation, confidence, physical competence, knowledge and understanding to value and take responsibility for engagement in physical activities for life” (International Physical Literacy Association, 2017). There were also several physical activity resources that did not focus on a specific topic (n = 17). The most common resource topics for sedentary behaviour were screen time (n = 5) and the Canadian 24-h Movement Guidelines (Tremblay et al., 2017b) (n = 11), which include screen time recommendations. Similarly, for sleep the Canadian 24-h Movement Guidelines (Tremblay et al., 2017b) (n = 11), which also include sleep recommendations, was the most common topic.

**Table 2:** Topics of included resources for each health behaviour

Nutrition		Physical activity		Sedentary behaviour		Sleep	
Topic	# of resources^a^	Topic	# of resources^a^	Topic	# of resources^a^	Topic	# of resources^a^
Menu/meal planning	55	Activities/Games	33	24-Hour Movement Guidelines	11	24-Hour Movement Guidelines	11
Healthy practices/environment	37	General	17	Screen time	5	Policy	1
Nutrition/food literacy	20	Physical literacy	13	General	2		
Recipes	15	Outdoor play	12	Policy	1		
General	9	Healthy practices/environment	11				
Food guide	6	24-Hour Movement Guidelines	11				
Breastfeeding	5	Loose parts	7				
Policy	3	Policy	7				
Indigenous food	2	Active play	3				
Family style meals	2	Lesson plans	3				
Fruit and vegetables	2	Dance	2				
Plant based diets	2	Indigenous games	1				
Healthy celebrations	1	Adapted physical activity	2				
		Cooperation through PA	1				

^a^The number of topics does not equal the number of included resources because some resources had more than one topic

### Quality of Resources

**Fig. 3:**
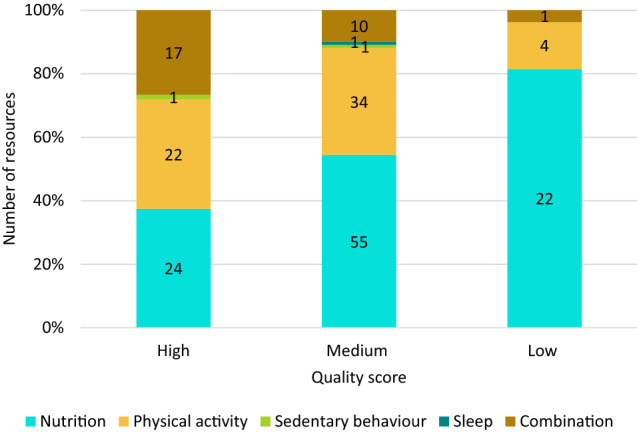
Quality of included resources stratified by health behaviour. The combination category includes resources that targeted more than one health behaviour

After resolving discrepancies, 27 resources were scored as low quality, 101 as medium quality, and 64 as high quality. The quality of resources stratified by health behaviour is displayed in Fig. 3. There was a similar proportion of high-quality resources targeting only nutrition (38%) or physical activity (34%). In comparison, most low-quality resources targeted only nutrition (81%). As a result, 40% of physical activity resources were high-quality compared to 22% of nutrition resources. Additionally, 61% of resources that targeted multiple behaviours were high-quality. Of the 64 high-quality resources, 55 resources received a point for the significance criterion (i.e., meaningful, unique, impactful), whereas only 13 resources received points for all 6 criteria. Of the 55 resources, all but five targeted nutrition (n = 21), physical activity (n = 20), or both health behaviours (n = 9), and the most common mode was a manual (n = 16). All 13 resources that received a score of 6 targeted nutrition (n = 6), physical activity (n = 6), or both health behaviours (n = 1), and the most common modes were manual (n = 4) and webinar (n = 4). Overall, many of these resources covered multiple topics but healthy practices/environment and menu/meal planning were the most common topics.

For inter-rater reliability the Cohen’s Kappa score was substantial for five criteria, including authority (К = 0.66), accuracy (К = 0.63), coverage (К = 0.66), objectivity (К = 0.92), and date (К = 0.96). However, for significance, inter-rater reliability was moderate (К = 0.50).

## Discussion

The objective of the study was to conduct an environmental scan of existing Canadian childcare resources targeting nutrition, physical activity, sedentary behaviour, and/or sleep. Using a comprehensive search plan that involved four search strategies, 192 eligible resources were identified. Although a number of included resources did not cite evidence and were undated, 64 high-quality resources were identified. In terms of health behaviours, the highest number of resources were found for nutrition followed by physical activity, combinations of health behaviours, sedentary behaviour, and sleep. However, the highest proportion of high-quality resources was found within the combination of health behaviours category, followed by physical activity and nutrition. This discussion section primarily focuses on high-quality resources throughout to highlight the best resources that currently exist and to inform future resource development with similar features.

A main finding of the environmental scan is that there are ample high-quality Canadian childcare resources for nutrition and physical activity, with some of these resources targeting both behaviours. Childcare centres are in a unique position to impact the quality and variety of food children eat because snacks and lunches are often provided daily in many centres (Larson et al., 2011; Ward et al., 2017). Childcare regulations in most Canadian provinces and territories stipulate that the Canada Food Guide should be followed, though specific recommendations regarding food and beverages are rare (Vercammen et al., 2020). As a result, research suggests improvements in the nutritional quality of foods is needed in care settings (Larson et al., 2011; Ward et al., 2017). This may explain why menu/meal planning was one of the most common resource topics across nutrition resources and of the highest quality resources. There were a wide variety of other nutrition topics covered with many targeting areas that research has suggested needs improvement in childcare settings (Larson et al., 2011). For instance, a number of resources and some of high-quality focused on healthy practices/environments, which covered subjects such as not forcing children to eat foods, having educators sit with children, and positive role modelling. Interestingly, provincial/territorial government and public health authorities were the most common source for nutrition resources. Therefore, government and related organizations may develop nutrition resources with more specific details to help support the implementation of broad nutrition regulations into practice (Vercammen et al., 2020). In addition, a number of included resources were based on the old food guide in Canada. Given the new food guide, which was released in 2019, takes a new holistic approach to healthy eating (Health Canada, 2019), comprehensive, high-quality resources will be needed that not only focus on what is being served but also on healthy practices and environments regarding nutrition.

Though fewer physical activity resources were found compared to nutrition, a similar amount of high-quality resources existed for both behaviours. Similar to nutrition, provincial/territorial childcare regulations in Canada regarding physical activity are primarily vague with general recommendations for gross motor movement and outdoor play (Vercammen et al., 2020). Additionally, few early child educator candidates in Canada receive formal training in physical activity (Bruijns et al., 2019; Martyniuk & Tucker, 2014). Therefore, physical activity childcare resources can play a major role in providing directors and educators with more specific details regarding this critical health behaviour. The most common resource topic was activities/games. These resources can provide educators with practical and age-appropriate tips and ideas for promoting physical activity and physical literacy, including the development of fundamental movement skills. Previous research has shown that educators want resources on this topic (Tucker et al., 2011). Outdoor play was also another common resource topic for physical activity. Previous studies have found children are more physically active when outdoors in childcare settings, compared to indoors (Raustorp et al., 2012; Tandon et al., 2018; Vanderloo et al., 2013). However, outdoor time permitted at childcare centres was found to be lower in the winter compared to non-winter months in one Canadian province (Predy et al., 2021). Many outdoor play resources touched on the importance of being active in all seasons and some resources provided specific example activities for outdoor play in the winter.

In contrast to nutrition, less government support in the form of resources was observed for physical activity. Given behaviour specific groups was the most common source of physical activity resources, childcare settings can benefit from experts in physical activity and outdoor play who have developed the resources. Additionally, across health behaviours, physical activity had the most resources that included the topic of policy. These resources can be used to create centre level policies that contain more specific recommendations regarding physical activity than those provided by provincial/territorial regulations. However, the majority of Canadian childcare centres do not have a written physical activity policy (Ott et al., 2019) and therefore centres may need additional support accessing and utilizing information found in policy focused resources. Overall, no major resource gaps were observed for topics related to physical activity.

Few resources focused on sedentary behaviour or sleep, except for resources that focused on the 24-h Movement Guidelines (Tremblay et al., 2017b). These guidelines provide the most up-to-date recommendations for physical activity, sedentary behaviour, and sleep (Tremblay et al., 2017b). However, the recommendations are for the entire 24-h period and are not specific to the childcare setting. Thus, many resources expanded on the recommendations to inform educators/directors of the importance of these behaviours for child development and some resources provided tips or ideas, specific to childcare settings. It is important to note that sleep tended to receive less attention than other health behaviours within the resources focusing on 24-h Movement Guidelines. Consequently, more Canadian sleep resources are needed for childcare settings. When developing these resources, potential partnerships with experts from sleep organizations and the use of modes that were identified for other high-quality resources in this environmental scan, such as manuals, online courses or modules, and webinars, should be considered. Additionally, given the links between sleep and other health behaviours and the finding that included resources targeting multiple behaviours tended to be of higher quality, new or updated resources may not want to solely focus on sleep. Future resources that incorporate sleep could focus on sleep hygiene or practices that can facilitate sound sleep (e.g., consistent routines, dark, quiet, and relaxing environments) (CSEP, 2017; Mindell et al., 2009). Another important topic to consider for future resources is accommodating different age and developmental requirements for regular extended nap periods in childcare settings (Staton et al., 2015b). Findings from a systematic review indicate that napping is associated with several aspects of night sleep in children older than 2 years of age, including later onset, lower sleep quality, and shorter duration (Thorpe et al., 2015). Specific to childcare settings, this association appears most salient for night sleep duration when nap or rest times are mandatory and for longer periods (Staton et al., 2015a). Given the direct implications of napping on nighttime sleep, resources should also consider how best to work in partnership with parents to improve this important health behaviour across home and childcare settings (Staton et al., 2015b).

Of the limited resources that focused on sedentary behaviour, a consistent message of limiting screen time was found. However, many Canadian children do not meet screen time recommendations (Carson et al., 2019; Chaput et al., 2017a; Lee et al., 2017) and childcare regulations for screen time is limited (Vercammen et al., 2020). It has previously been reported that access and use of screens are prevalent in many childcare settings internationally (Vanderloo, 2014). Therefore, it appears additional high-quality Canadian screen time resources are needed for childcare settings. For example, systematic review evidence indicates high staff education is a consistent negative correlate of screen time in childcare settings (Vanderloo, 2014). Therefore, professional development resources using assessable modes that were identified for other high-quality resources, such as online courses or modules, may be particularly important. Given, that screen time tends to be higher at home compared to in care (Tandon et al., 2011), additional family education resources providing practical tips and ideas (Carson et al., 2014) that can be accessed through childcare settings may also be beneficial.

This environmental scan represents an important first step in intervention planning for childcare settings by identifying high-quality and applicable resources to assist this work. By only including resources that targeted improvements in behaviours, we purposely addressed the “how” to promote healthy behavioural patterns versus just the “why” it is important to promote healthy behavioural patterns, which may have a more meaningful impact (Arlinghaus & Johnston, 2017). Additionally, some resources in our environmental scan went beyond individual-level factors to consider environments, partnerships, and policy. This is important as evidence from a scoping review indicates the theories, models, and frameworks used in interventions targeting nutrition in childcare centres tend to focus on the individual-level of the childcare provider (Lima do Vale et al., 2020), therefore missing important institutional/organizational, community/environmental, and policy-related factors of the social ecological model (Golden & Earp, 2012). It is important to note, education alone is not effective in changing behaviours (Arlinghaus & Johnston, 2017). Therefore, the findings of this scan can inform, support, and complement future planning steps related to interventions in childcare settings. Though there will be a time lag between when this environmental scan was conducted and the subsequent implementation of an intervention in childcare settings, the vast majority of resources identified will still be applicable in the future. Additionally, the identification of key organizations will enable the efficient identification of new resources in the future. The findings of this environmental scan also have important implications beyond research interventions. Most importantly, the database of resources developed can be utilized by stakeholders in the childcare sector.

The main strength of this environmental scan was the comprehensive search plan that followed an established template, which was reviewed by a librarian with expertise in systematic reviews. Additional strengths are the inclusion of multiple health behaviours and the quality assessment of included resources. A limitation of the environmental scan was the focus on only Canadian resources. However, without this limit, the scan would likely have not been feasible in scope. Additionally, this scan is part of a larger project that will take place in Canada.

## Conclusion

This environmental scan identified several high-quality resources specific to childcare settings that target these health behaviours as well as gaps that should be filled with future resource development. To ensure future resources are high quality, up-to-date evidence should be utilized to inform them and resources should be dated so it is clear how current they are. Overall, findings can support future health promotion interventions in childcare settings. Additionally, the database of resources generated can support other childcare initiatives aiming to promote healthy behavioural patterns in early childhood.

## Supplementary Information

Below is the link to the electronic supplementary material.

Supplementary file1 (DOCX 21 kb)

Supplementary file2 (DOCX 13 kb)

Supplementary file3 (DOCX 23 kb)

Supplementary file4 (XLSX 49 kb)

## Data Availability

Not applicable.
