# Supplementary material for: An Environmental Scan of Existing Canadian Childcare Resources Targeting Improvements in Health Behaviours
Source: Early Child Educ J. 2021 Sep 28;50(8):1417–28. doi: 10.1007/s10643-021-01266-2 (PMC9622543; doi:10.1007/s10643-021-01266-2)
Supplement: Supplementary file 1 — Supplementary file1 (DOCX 21 kb) [file 10643_2021_1266_MOESM1_ESM.docx]

**Supplementary File 1: Terms used for Grey Literature Database Search**

| # | Search Term |
| --- | --- |
| 1 | "physical activity" AND "child care" AND "resources" |
| 2 | "physical activity" AND "child care" AND "standards" |
| 3 | "physical activity" AND "child care" AND "curriculum" |
| 4 | "physical activity" AND "child care" AND "recommendations" |
| 5 | "physical activity" AND "child care" AND "guidelines" |
| 6 | "physical activity" AND "child care" AND "policies" |
| 7 | "physical activity" AND "child care" AND "best practices" |
| 8 | "physical activity" AND "child care" AND "activities" |
| 9 | "physical activity" AND "child care" AND "tool kit" |
| 10 | "physical activity" AND “daycare” AND "resources" |
| 11 | "physical activity" AND “daycare” AND "standards" |
| 12 | "physical activity" AND “daycare” AND "curriculum" |
| 13 | "physical activity" AND “daycare” AND "recommendations" |
| 14 | "physical activity" AND “daycare” AND "guidelines" |
| 15 | "physical activity" AND “daycare” AND "policies" |
| 16 | "physical activity" AND “daycare” AND "best practices" |
| 17 | "physical activity" AND “daycare” AND "activities" |
| 18 | "physical activity" AND “daycare” AND "tool kit" |
| 19 | “gross motor” AND "child care" AND "resources" |
| 20 | “gross motor” AND "child care" AND "standards" |
| 21 | “gross motor” AND "child care" AND "curriculum" |
| 22 | “gross motor” AND "child care" AND "recommendations" |
| 23 | “gross motor” AND "child care" AND "guidelines" |
| 24 | “gross motor” AND "child care" AND "policies" |
| 25 | “gross motor” AND "child care" AND "best practices" |
| 26 | “gross motor” AND "child care" AND "activities" |
| 27 | “gross motor” AND "child care" AND "tool kit" |
| 28 | “gross motor” AND “daycare” AND "resources" |
| 29 | “gross motor” AND “daycare” AND "standards" |
| 30 | “gross motor” AND “daycare” AND "curriculum" |
| 31 | “gross motor” AND “daycare” AND "recommendations" |
| 32 | “gross motor” AND “daycare” AND "guidelines" |
| 33 | “gross motor” AND “daycare” AND "policies" |
| 34 | “gross motor” AND “daycare” AND "best practices" |
| 35 | “gross motor” AND “daycare” AND "activities" |
| 36 | “gross motor” AND “daycare” AND "tool kit" |
| 37 | “outdoor” AND "child care" AND "resources" |
| 38 | “outdoor” AND "child care" AND "standards" |
| 39 | “outdoor” AND "child care" AND "curriculum" |
| 40 | “outdoor” AND "child care" AND "recommendations" |
| 41 | “outdoor” AND "child care" AND "guidelines" |
| 42 | “outdoor” AND "child care" AND "policies" |
| 43 | “outdoor” AND "child care" AND "best practices" |
| 44 | “outdoor” AND "child care" AND "activities" |
| 45 | “outdoor” AND "child care" AND "tool kit" |
| 46 | “outdoor” AND “daycare” AND "resources" |
| 47 | “outdoor” AND “daycare” AND "standards" |
| 48 | “outdoor” AND “daycare” AND "curriculum" |
| 49 | “outdoor” AND “daycare” AND "recommendations" |
| 50 | “outdoor” AND “daycare” AND "guidelines" |
| 51 | “outdoor” AND “daycare” AND "policies" |
| 52 | “outdoor” AND “daycare” AND "best practices" |
| 53 | “outdoor” AND “daycare” AND "activities" |
| 54 | “outdoor” AND “daycare” AND "tool kit" |
| 55 | “physical literacy” AND "child care" AND "resources" |
| 56 | “physical literacy” AND "child care" AND "standards" |
| 57 | “physical literacy” AND "child care" AND "curriculum" |
| 58 | “physical literacy” AND "child care" AND "recommendations" |
| 59 | “physical literacy” AND "child care" AND "guidelines" |
| 60 | “physical literacy” AND "child care" AND "policies" |
| 61 | “physical literacy” AND "child care" AND "best practices" |
| 62 | “physical literacy” AND "child care" AND "activities" |
| 63 | “physical literacy” AND "child care" AND "tool kit" |
| 64 | “physical literacy” AND “daycare” AND "resources" |
| 65 | “physical literacy” AND “daycare” AND "standards" |
| 66 | “physical literacy” AND “daycare” AND "curriculum" |
| 67 | “physical literacy” AND “daycare” AND "recommendations" |
| 68 | “physical literacy” AND “daycare” AND "guidelines" |
| 69 | “physical literacy” AND “daycare” AND "policies" |
| 70 | “physical literacy” AND “daycare” AND "best practices" |
| 71 | “physical literacy” AND “daycare” AND "activities" |
| 72 | “physical literacy” AND “daycare” AND "tool kit" |
| 73 | “sedentary behaviour” AND "child care" AND "resources" |
| 74 | “sedentary behaviour” AND "child care" AND "standards" |
| 75 | “sedentary behaviour” AND "child care" AND "curriculum" |
| 76 | “sedentary behaviour” AND "child care" AND "recommendations" |
| 77 | “sedentary behaviour” AND "child care" AND "guidelines" |
| 78 | “sedentary behaviour” AND "child care" AND "policies" |
| 79 | “sedentary behaviour” AND "child care" AND "best practices" |
| 80 | “sedentary behaviour” AND "child care" AND "activities" |
| 81 | “sedentary behaviour” AND "child care" AND "tool kit" |
| 82 | “sedentary behaviour” AND “daycare” AND "resources" |
| 83 | “sedentary behaviour” AND “daycare” AND "standards" |
| 84 | “sedentary behaviour” AND “daycare” AND "curriculum" |
| 85 | “sedentary behaviour” AND “daycare” AND "recommendations" |
| 86 | “sedentary behaviour” AND “daycare” AND "guidelines" |
| 87 | “sedentary behaviour” AND “daycare” AND "policies" |
| 88 | “sedentary behaviour” AND “daycare” AND "best practices" |
| 89 | “sedentary behaviour” AND “daycare” AND "activities" |
| 90 | “sedentary behaviour” AND “daycare” AND "tool kit" |
| 91 | “screen time” AND "child care" AND "resources" |
| 92 | “screen time” AND "child care" AND "standards" |
| 93 | “screen time” AND "child care" AND "curriculum" |
| 94 | “screen time” AND "child care" AND "recommendations" |
| 95 | “screen time” AND "child care" AND "guidelines" |
| 96 | “screen time” AND "child care" AND "policies" |
| 97 | “screen time” AND "child care" AND "best practices" |
| 98 | “screen time” AND "child care" AND "activities" |
| 99 | “screen time” AND "child care" AND "tool kit" |
| 100 | “screen time” AND “daycare” AND "resources" |
| 101 | “screen time” AND “daycare” AND "standards" |
| 102 | “screen time” AND “daycare” AND "curriculum" |
| 103 | “screen time” AND “daycare” AND "recommendations" |
| 104 | “screen time” AND “daycare” AND "guidelines" |
| 105 | “screen time” AND “daycare” AND "policies" |
| 106 | “screen time” AND “daycare” AND "best practices" |
| 107 | “screen time” AND “daycare” AND "activities" |
| 108 | “screen time” AND “daycare” AND "tool kit" |
| 109 | “television” AND "child care" AND "resources" |
| 110 | “television” AND "child care" AND "standards" |
| 111 | “television” AND "child care" AND "curriculum" |
| 112 | “television” AND "child care" AND "recommendations" |
| 113 | “television” AND "child care" AND "guidelines" |
| 114 | “television” AND "child care" AND "policies" |
| 115 | “television” AND "child care" AND "best practices" |
| 116 | “television” AND "child care" AND "activities" |
| 117 | “television” AND "child care" AND "tool kit" |
| 118 | “television” AND “daycare” AND "resources" |
| 119 | “television” AND “daycare” AND "standards" |
| 120 | “television” AND “daycare” AND "curriculum" |
| 121 | “television” AND “daycare” AND "recommendations" |
| 122 | “television” AND “daycare” AND "guidelines" |
| 123 | “television” AND “daycare” AND "policies" |
| 124 | “television” AND “daycare” AND "best practices" |
| 125 | “television” AND “daycare” AND "activities" |
| 126 | “television” AND “daycare” AND "tool kit" |
| 127 | “computer” AND "child care" AND "resources" |
| 128 | “computer” AND "child care" AND "standards" |
| 129 | “computer” AND "child care" AND "curriculum" |
| 130 | “computer” AND "child care" AND "recommendations" |
| 131 | “computer” AND "child care" AND "guidelines" |
| 132 | “computer” AND "child care" AND "policies" |
| 133 | “computer” AND "child care" AND "best practices" |
| 134 | “computer” AND "child care" AND "activities" |
| 135 | “computer” AND "child care" AND "tool kit" |
| 136 | “computer” AND “daycare” AND "resources" |
| 137 | “computer” AND “daycare” AND "standards" |
| 138 | “computer” AND “daycare” AND "curriculum" |
| 139 | “computer” AND “daycare” AND "recommendations" |
| 140 | “computer” AND “daycare” AND "guidelines" |
| 141 | “computer” AND “daycare” AND "policies" |
| 142 | “computer” AND “daycare” AND "best practices" |
| 143 | “computer” AND “daycare” AND "activities" |
| 144 | “computer” AND “daycare” AND "tool kit" |
| 145 | “media” AND "child care" AND "resources" |
| 146 | “media” AND "child care" AND "standards" |
| 147 | “media” AND "child care" AND "curriculum" |
| 148 | “media” AND "child care" AND "recommendations" |
| 149 | “media” AND "child care" AND "guidelines" |
| 150 | “media” AND "child care" AND "policies" |
| 151 | “media” AND "child care" AND "best practices" |
| 152 | “media” AND "child care" AND "activities" |
| 153 | “media” AND "child care" AND "tool kit" |
| 154 | “media” AND “daycare” AND "resources" |
| 155 | “media” AND “daycare” AND "standards" |
| 156 | “media” AND “daycare” AND "curriculum" |
| 157 | “media” AND “daycare” AND "recommendations" |
| 158 | “media” AND “daycare” AND "guidelines" |
| 159 | “media” AND “daycare” AND "policies" |
| 160 | “media” AND “daycare” AND "best practices" |
| 161 | “media” AND “daycare” AND "activities" |
| 162 | “media” AND “daycare” AND "tool kit" |
| 163 | “sleep” AND "child care" AND "resources" |
| 164 | “sleep” AND "child care" AND "standards" |
| 165 | “sleep” AND "child care" AND "curriculum" |
| 166 | “sleep” AND "child care" AND "recommendations" |
| 167 | “sleep” AND "child care" AND "guidelines" |
| 168 | “sleep” AND "child care" AND "policies" |
| 169 | “sleep” AND "child care" AND "best practices" |
| 170 | “sleep” AND "child care" AND "activities" |
| 171 | “sleep” AND "child care" AND "tool kit" |
| 172 | “sleep” AND “daycare” AND "resources" |
| 173 | “sleep” AND “daycare” AND "standards" |
| 174 | “sleep” AND “daycare” AND "curriculum" |
| 175 | “sleep” AND “daycare” AND "recommendations" |
| 176 | “sleep” AND “daycare” AND "guidelines" |
| 177 | “sleep” AND “daycare” AND "policies" |
| 178 | “sleep” AND “daycare” AND "best practices" |
| 179 | “sleep” AND “daycare” AND "activities" |
| 180 | “sleep” AND “daycare” AND "tool kit" |
| 181 | “nap” AND "child care" AND "resources" |
| 182 | “nap” AND "child care" AND "standards" |
| 183 | “nap” AND "child care" AND "curriculum" |
| 184 | “nap” AND "child care" AND "recommendations" |
| 185 | “nap” AND "child care" AND "guidelines" |
| 186 | “nap” AND "child care" AND "policies" |
| 187 | “nap” AND "child care" AND "best practices" |
| 188 | “nap” AND "child care" AND "activities" |
| 189 | “nap” AND "child care" AND "tool kit" |
| 190 | “nap” AND “daycare” AND "resources" |
| 191 | “nap” AND “daycare” AND "standards" |
| 192 | “nap” AND “daycare” AND "curriculum" |
| 193 | “nap” AND “daycare” AND "recommendations" |
| 194 | “nap” AND “daycare” AND "guidelines" |
| 195 | “nap” AND “daycare” AND "policies" |
| 196 | “nap” AND “daycare” AND "best practices" |
| 197 | “nap” AND “daycare” AND "activities" |
| 198 | “nap” AND “daycare” AND "tool kit" |
| 199 | “rest” AND "child care" AND "resources" |
| 200 | “rest” AND "child care" AND "standards" |
| 201 | “rest” AND "child care" AND "curriculum" |
| 202 | “rest” AND "child care" AND "recommendations" |
| 203 | “rest” AND "child care" AND "guidelines" |
| 204 | “rest” AND "child care" AND "policies" |
| 205 | “rest” AND "child care" AND "best practices" |
| 206 | “rest” AND "child care" AND "activities" |
| 207 | “rest” AND "child care" AND "tool kit" |
| 208 | “rest” AND “daycare” AND "resources" |
| 209 | “rest” AND “daycare” AND "standards" |
| 210 | “rest” AND “daycare” AND "curriculum" |
| 211 | “rest” AND “daycare” AND "recommendations" |
| 212 | “rest” AND “daycare” AND "guidelines" |
| 213 | “rest” AND “daycare” AND "policies" |
| 214 | “rest” AND “daycare” AND "best practices" |
| 215 | “rest” AND “daycare” AND "activities" |
| 216 | “rest” AND “daycare” AND "tool kit" |
| 217 | “nutrition” AND "child care" AND "resources" |
| 218 | “nutrition” AND "child care" AND "standards" |
| 219 | “nutrition” AND "child care" AND "curriculum" |
| 220 | “nutrition” AND "child care" AND "recommendations" |
| 221 | “nutrition” AND "child care" AND "guidelines" |
| 222 | “nutrition” AND "child care" AND "policies" |
| 223 | “nutrition” AND "child care" AND "best practices" |
| 224 | “nutrition” AND "child care" AND "activities" |
| 225 | “nutrition” AND "child care" AND "tool kit" |
| 226 | “nutrition” AND “daycare” AND "resources" |
| 227 | “nutrition” AND “daycare” AND "standards" |
| 228 | “nutrition” AND “daycare” AND "curriculum" |
| 229 | “nutrition” AND “daycare” AND "recommendations" |
| 230 | “nutrition” AND “daycare” AND "guidelines" |
| 231 | “nutrition” AND “daycare” AND "policies" |
| 232 | “nutrition” AND “daycare” AND "best practices" |
| 233 | “nutrition” AND “daycare” AND "activities" |
| 234 | “nutrition” AND “daycare” AND "tool kit" |
| 235 | “healthy eating” AND "child care" AND "resources" |
| 236 | “healthy eating” AND "child care" AND "standards" |
| 237 | “healthy eating” AND "child care" AND "curriculum" |
| 238 | “healthy eating” AND "child care" AND "recommendations" |
| 239 | “healthy eating” AND "child care" AND "guidelines" |
| 240 | “healthy eating” AND "child care" AND "policies" |
| 241 | “healthy eating” AND "child care" AND "best practices" |
| 242 | “healthy eating” AND "child care" AND "activities" |
| 243 | “healthy eating” AND "child care" AND "tool kit" |
| 244 | “healthy eating” AND “daycare” AND "resources" |
| 245 | “healthy eating” AND “daycare” AND "standards" |
| 246 | “healthy eating” AND “daycare” AND "curriculum" |
| 247 | “healthy eating” AND “daycare” AND "recommendations" |
| 248 | “healthy eating” AND “daycare” AND "guidelines" |
| 249 | “healthy eating” AND “daycare” AND "policies" |
| 250 | “healthy eating” AND “daycare” AND "best practices" |
| 251 | “healthy eating” AND “daycare” AND "activities" |
| 252 | “healthy eating” AND “daycare” AND "tool kit" |
| 253 | “snack” AND "child care" AND "resources" |
| 254 | “snack” AND "child care" AND "standards" |
| 255 | “snack” AND "child care" AND "curriculum" |
| 256 | “snack” AND "child care" AND "recommendations" |
| 257 | “snack” AND "child care" AND "guidelines" |
| 258 | “snack” AND "child care" AND "policies" |
| 259 | “snack” AND "child care" AND "best practices" |
| 260 | “snack” AND "child care" AND "activities" |
| 261 | “snack” AND "child care" AND "tool kit" |
| 262 | “snack” AND “daycare” AND "resources" |
| 263 | “snack” AND “daycare” AND "standards" |
| 264 | “snack” AND “daycare” AND "curriculum" |
| 265 | “snack” AND “daycare” AND "recommendations" |
| 266 | “snack” AND “daycare” AND "guidelines" |
| 267 | “snack” AND “daycare” AND "policies" |
| 268 | “snack” AND “daycare” AND "best practices" |
| 269 | “snack” AND “daycare” AND "activities" |
| 270 | “snack” AND “daycare” AND "tool kit" |
| 271 | “lunch” AND "child care" AND "resources" |
| 272 | “lunch” AND "child care" AND "standards" |
| 273 | “lunch” AND "child care" AND "curriculum" |
| 274 | “lunch” AND "child care" AND "recommendations" |
| 275 | “lunch” AND "child care" AND "guidelines" |
| 276 | “lunch” AND "child care" AND "policies" |
| 277 | “lunch” AND "child care" AND "best practices" |
| 278 | “lunch” AND "child care" AND "activities" |
| 279 | “lunch” AND "child care" AND "tool kit" |
| 280 | “lunch” AND “daycare” AND "resources" |
| 281 | “lunch” AND “daycare” AND "standards" |
| 282 | “lunch” AND “daycare” AND "curriculum" |
| 283 | “lunch” AND “daycare” AND "recommendations" |
| 284 | “lunch” AND “daycare” AND "guidelines" |
| 285 | “lunch” AND “daycare” AND "policies" |
| 286 | “lunch” AND “daycare” AND "best practices" |
| 287 | “lunch” AND “daycare” AND "activities" |
| 288 | “lunch” AND “daycare” AND "tool kit" |

**Note:**Six databases were searched using the above terms. Specific search dates are noted below. Where specified, the database used “OR” to connect terms instead of “AND”.
- Canadian Agency for Drugs and Technologies in Health (CADTH) searched Sept 19-24, 2019
- Canadian Best Practices Portal searched Sept 24, 2019; using “OR”
- Health Systems Evidence searched Sept 24, 2019
- Canadian Health & Human Resources Network (CHHRN) searched Sept 24, 2019; using “OR”
- Public Health Grey Literature Database searched Sept 25, 2019
- Turing Research Into Practice (TRIP) Medical Database searched Oct7-10, 2019
