# Supplementary material for: An Environmental Scan of Existing Canadian Childcare Resources Targeting Improvements in Health Behaviours
Source: Early Child Educ J. 2021 Sep 28;50(8):1417–28. doi: 10.1007/s10643-021-01266-2 (PMC9622543; doi:10.1007/s10643-021-01266-2)
Supplement: Supplementary file 2 — Supplementary file2 (DOCX 13 kb) [file 10643_2021_1266_MOESM2_ESM.docx]

**Supplementary File 2: Terms used for Customized Google Search Engines and Targeted Websites Search**

| # | Search Term |
| --- | --- |
| 1 | “physical activity" AND “child care” OR “day care” AND resources |
| 2 | “physical activity" AND “child care” OR “day care” AND recommendations guidelines policies “best practices” |
| 3 | “physical activity" AND “child care” OR “day care” AND standards curriculum |
| 4 | “physical activity" AND “child care” OR “day care” AND activities “tool kit” |
| 5 | “child care” AND “gross motor” outdoor “physical literacy” AND resources |
| 6 | “child care” AND “gross motor” outdoor “physical literacy” AND recommendations guidelines policies “best practices” |
| 7 | “child care” AND “gross motor” outdoor “physical literacy” AND standards curriculum |
| 8 | “child care”” AND “gross motor” outdoor “physical literacy” AND activities “tool kit” |
| 9 | “screen time” AND “child care” OR “day care” AND resources |
| 10 | “screen time” AND “child care” OR “day care” AND recommendations guidelines policies “best practices” |
| 11 | “screen time” AND “child care” OR “day care” AND standards curriculum |
| 12 | “screen time" AND “child care” OR “day care” AND activities “tool kit” |
| 13 | “child care” AND television computer media AND resources |
| 14 | “child care” AND television computer media AND recommendations guidelines policies “best practices” |
| 15 | “child care” AND television computer media AND standards curriculum |
| 16 | “child care”” AND television computer media AND activities “tool kit” |
| 17 | sleep nap rest AND “child care” OR “day care” AND resources |
| 18 | sleep nap rest AND “child care” OR “day care” AND recommendations guidelines policies “best practices” |
| 19 | sleep nap rest AND “child care” OR “day care” AND standards curriculum |
| 20 | sleep nap rest AND “child care” OR “day care” AND activities “tool kit” |
| 21 | nutrition AND “child care” OR “day care” AND resources |
| 22 | nutrition AND “child care” OR “day care” AND recommendations guidelines policies “best practices” |
| 23 | nutrition AND “child care” OR “day care” AND standards curriculum |
| 24 | nutrition AND “child care” OR “day care” AND activities “tool kit” |
| 25 | “child care” AND “healthy eating” snack lunch AND resources |
| 26 | “child care” AND “healthy eating” snack lunch AND recommendations guidelines policies “best practices” |
| 27 | “child care” AND “healthy eating” snack lunch AND standards curriculum |
| 28 | “child care”” AND “healthy eating” snack lunch AND activities “tool kit” |

**Note:**Targeted websites were searched from Nov 4, 2019-March 24, 2020
Three customized Google search engines were searched using the above terms. Specific search dates are noted below.  **-** Canadian Public Health Associations searched Sept 13-19, 2019 **-** Ontario Public Health Unit Websites searched Sept 20-27, 2019 **-** Canadian Public Health Information* searched Oct 17- Nov 21, 2019
*The list of sites from this search engine was separated into smaller searches and done manually as the search engine was not functioning properly.
