# Supplementary material for: An Environmental Scan of Existing Canadian Childcare Resources Targeting Improvements in Health Behaviours
Source: Early Child Educ J. 2021 Sep 28;50(8):1417–28. doi: 10.1007/s10643-021-01266-2 (PMC9622543; doi:10.1007/s10643-021-01266-2)
Supplement: Supplementary file 3 — Supplementary file3 (DOCX 23 kb) [file 10643_2021_1266_MOESM3_ESM.docx]

**Supplementary File 3: Websites Identified from Targeted Website Searches**

| # | Organization/Website Name | Main URL |
| --- | --- | --- |
| 1 | Active for Life | https://activeforlife.com/ |
| 2 | Affiliated Services for Children and Youth | https://ascy.ca/ |
| 3 | Alberta Association for the Accreditation of Early Learning and Care Services | *link no longer available* |
| 4 | Alberta Education - Childcare framework | https://education.alberta.ca/ |
| 5 | Alberta Health Services | https://www.albertahealthservices.ca/ |
| 6 | Alberta Public Health Association | https://www.apha.ab.ca/ |
| 7 | Alberta Resource Center for Quality Enhancement | https://arcqe.ca/ |
| 8 | Algoma Public Health | http://www.algomapublichealth.com/ |
| 9 | Andrew Fleck Children's Services | https://www.afchildrensservices.ca/ |
| 10 | Association of Early Childhood Educators of Alberta | https://aecea.ca/ |
| 11 | Association of Day Care Operators of Ontario (ADCO) | https://adco-o.on.ca/ |
| 12 | Association of Early Childhood Educators NL | https://www.aecenl.ca/ |
| 13 | Association of Early Childhood Educators Ontario | https://www.aeceo.ca/ |
| 14 | Association of Local Public Health Agencies (alPHa) | https://www.alphaweb.org/ |
| 15 | BC Aboriginal Child Care Society | https://www.acc-society.bc.ca/ |
| 16 | BC Health Planning | http://health.gov.bc.ca/library/ |
| 17 | Be Fit for Life | http://befitforlife.ca/ |
| 18 | Best Start | https://en.beststart.org/ |
| 19 | Boys and Girls Club Niagara | https://www.boysandgirlsclubniagara.org/ |
| 20 | Burnaby Community & Continuing Education | https://www.burnabycce.ca/ |
| 21 | Canadian Child Care Association | *link no longer available* |
| 22 | Canadian Child Care Federation | https://www.cccf-fcsge.ca/ |
| 23 | Canadian Fitness & Lifestyle Research Institute | https://cflri.ca/ |
| 24 | Canadian Paediatric Society | https://www.cps.ca/ |
| 25 | Canadian Public Health Association | https://www.cpha.ca/ |
| 26 | Canadian Task Force on Preventive Health Care | https://canadiantaskforce.ca/ |
| 27 | Canchild | https://www.canchild.ca/ |
| 28 | Caring for kids | https://www.caringforkids.cps.ca/ |
| 29 | Centre for Active Living | https://www.centre4activeliving.ca/ |
| 30 | Centre of Excellence for Early Childhood Development | https://www.casw-acts.ca/en/ |
| 31 | Child and Youth Network | https://www.londoncyn.ca/ |
| 32 | Child care Algoma | https://childcarealgoma.ca/ |
| 33 | Child Care Canada – Child Care Resource and Research Unit | https://childcarecanada.org/ |
| 34 | Child Care Human Resources Sector Council | http://www.ccsc-cssge.ca/ |
| 35 | Child Care Providers Resource Network | https://ccprn.com/ |
| 36 | Child Care Resource & Referral | https://www.childcareoptions.ca/ |
| 37 | Child Care Solutions | https://childcaresolutions.ca/ |
| 38 | Child Care Victoria | https://www.childcarevictoria.org/ |
| 39 | Child Health and Exercise Med Program- McMaster University | https://fhs.mcmaster.ca/chemp/ |
| 40 | Child Health Grey Literature | *link no longer available* |
| 41 | Childcare Options | https://www.childcareoptions.ca/ |
| 42 | City of Hamilton | https://www.hamilton.ca/ |
| 43 | City of Windsor | https://www.citywindsor.ca/ |
| 44 | CMAS Canada | https://cmascanada.ca/ |
| 45 | College of the North Atlantic | https://www.cna.nl.ca/ |
| 46 | County of Welllington | https://www.wellington.ca/en/ |
| 47 | Creative Childcare Consulting | https://creativechildcareconsulting.ca/ |
| 48 | Cree Board of Health and Social Services of James Bay | https://creehealth.org/ |
| 49 | Davenport-Perth Neighbourhood and Community Health Centre | http://dpnchc.com/ |
| 50 | Decoda | https://www.decoda.ca/ |
| 51 | Delta Kids | https://www.deltakids.ca/ |
| 52 | Dieticians of Canada | https://www.dietitians.ca/ |
| 53 | Durham region | https://www.durham.ca/en/ |
| 54 | Early Childhood Care & Education New Brunswick | https://eccenb-sepenb.ca/en/ |
| 55 | Early Childhood Community Development Centre | https://eccdc.org/ |
| 56 | Early Childhood Development Association of PEI | https://www.ecdaofpei.ca/ |
| 57 | Early Childhood Educators of British Columbia | https://www.ecebc.ca/ |
| 58 | Early Learning and Child care Quality by Design | https://www.childcarequality.ca/ |
| 59 | EarlyON Child and Family Centre | https://earlyyearsinfo.ca/ |
| 60 | Early Years Physical Literacy | https://www.earlyyearsphysicalliteracy.com/ |
| 61 | East Kootenay Child Care Resource and Referral | http://ccrr.ccscranbrook.ca/ |
| 62 | Edmonton Early Years Coalitions | https://ecdcoalitions.org/coalition/edmonton-early-years-coalitions/ |
| 63 | Edmonton Sport Council | https://www.edmontonsport.com/ |
| 64 | Employment and Social Development Canada | https://www.canada.ca/en/employment-social-development.html |
| 65 | Encyclopedia on Early Childhood Development | http://www.child-encyclopedia.com/ |
| 66 | Family Day | https://familydaycare.com/ |
| 67 | Fraser Health | https://www.fraserhealth.ca/ |
| 68 | Get Outside and Play | https://getoutsideandplay.ca/ |
| 69 | Government of Alberta | https://www.alberta.ca/ |
| 70 | Government of BC | https://www2.gov.bc.ca/ |
| 71 | Government of Manitoba | https://www.gov.mb.ca/ |
| 72 | Government of New Brunswick | https://www2.gnb.ca/ |
| 73 | Government of Newfoundland and Labrador | https://www.gov.nl.ca/ |
| 74 | Government of Northwest Territories | https://www.gov.nt.ca/ |
| 75 | Government of Nova Scotia | https://novascotia.ca/ |
| 76 | Government of Nunavut | https://www.gov.nu.ca/ |
| 77 | Government of Ontario | https://www.ontario.ca/ |
| 78 | Government of Prince Edward Island | https://www.princeedwardisland.ca/en |
| 79 | Government of Saskatchewan | https://www.saskatchewan.ca/ |
| 80 | Government of Yukon | https://yukon.ca/ |
| 81 | Greater Sudbury | https://www.greatersudbury.ca/ |
| 82 | Haldimand-Norfolk Health Unit | https://hnhu.org/ |
| 83 | Halton Early Years Mental Health Committee - Our Kids Network | https://www.ourkidsnetwork.ca/ |
| 84 | Halton Region | https://www.halton.ca/ |
| 85 | Hastings Prince Edward Public Health | https://hpepublichealth.ca/ |
| 86 | Have a Ball Together! | https://haveaballtogether.ca/ |
| 87 | Health and Social Services Haldimand and Norfolk | https://www.haldimandcounty.ca/ |
| 88 | Health Link BC | https://www.healthlinkbc.ca/ |
| 89 | Healthy Active Preschoolers | *link no longer available* |
| 90 | Healthy Beginnings for Preschoolers | https://www.healthybeginningspreschoolers.ca/ |
| 91 | Healthy Child Manitoba | https://www.gov.mb.ca/healthychild/ |
| 92 | Healthy Populations Institute | https://www.healthypopulationsinstitute.ca/ |
| 93 | Healthy Start | https://healthystartkids.ca/ |
| 94 | Healthy Eating Physical Activity Coalition of New Brunswick (HEPAC) | https://hepac.ca/ |
| 95 | Institut national de santé publique (INSPQ) | https://www.inspq.qc.ca/en |
| 96 | Interior Health | https://www.interiorhealth.ca/ |
| 97 | Kingston, Frontenac and Lennox & Addington Public Health (KFLA) | https://www.kflaph.ca/en/ |
| 98 | Lawson Foundation | https://lawson.ca/ |
| 99 | Leeds Grenville & Lanark District Health Unit | https://healthunit.org/ |
| 100 | LIVE 5210 | https://www.live5210.ca/ |
| 101 | Manitoba Child Care Association | http://mccahouse.org/ |
| 102 | Manitoba Public Health Association | https://manitobapha.ca/ |
| 103 | Maternal and Child Health Information Resource Centre | https://mchb.hrsa.gov/ |
| 104 | Middlesex County | https://www.middlesex.ca/ |
| 105 | Middlesex-London Health Unit | https://www.healthunit.com/ |
| 106 | Mount Saint Vincent University | https://www.msvu.ca/ |
| 107 | Moving to Learn | http://movingtolearn.ca/ |
| 108 | Nemours Children's Health System | https://www.nemours.org/ |
| 109 | New Brunswick Plays | https://www.nbplays.ca/the-resources |
| 110 | North Bay Parry Sound District Health Unit | https://www.myhealthunit.ca/ |
| 111 | North Edmonton Family Day Home Agency | http://nefdha.com/ |
| 112 | Northern Health | https://www.northernhealth.ca/ |
| 113 | Northwest Territories (Education, Culture, and Employment) | https://www.ece.gov.nt.ca/en |
| 114 | Nova Scotia Child Care Association | https://aecens.ca/ |
| 115 | Nutrition Resource Centre | https://opha.on.ca/Nutrition-Resource-Centre/ |
| 116 | Ontario Dietitians in Public Health | https://www.odph.ca/ |
| 117 | Ontario Health Promotion e-Bulletin (children focus) | http://www.ohpe.ca/taxonomy/term/71 |
| 118 | Ontario Ministry of Education | http://www.edu.gov.on.ca/earlyyears/ |
| 119 | Ontario Public Health Association | https://opha.on.ca/ |
| 120 | Ontario Society of Nutrition Professionals in Public Health | https://www.osnpph.on.ca/ |
| 121 | OPHEA Healthy Schools Healthy Communities | https://www.ophea.net/ |
| 122 | Ottawa Public Health | https://www.ottawapublichealth.ca/en/index.aspx |
| 123 | ParticpACTION | https://www.participaction.com/en-ca |
| 124 | Peel District School Board | https://www.peelschools.org/ |
| 125 | PEI Healthy Eating Alliance and PEI Department of Education and Early Childhood Development | http://www.gov.pe.ca/photos/original/eecd_healthyliv.pdf |
| 126 | Peterborough Public Health | https://www.peterboroughpublichealth.ca/ |
| 127 | Porcupine Health Unit | https://www.porcupinehu.on.ca/en/ |
| 128 | Public Health - Wellington - Dufferin - Guelph | https://www.wdgpublichealth.ca/ |
| 129 | Public Health Agency of Canada | https://www.canada.ca/en/public-health |
| 130 | Public Health Association of Nova Scotia | https://www.phans.ca/ |
| 131 | Public Health Grey Bruce Health Unit | https://www.publichealthgreybruce.on.ca/ |
| 132 | Public Health Ontario | https://www.publichealthontario.ca/ |
| 133 | Public Health- Sudbury & Districts | https://www.phsd.ca/ |
| 134 | Real food for Real Kids | https://www.rfrk.com/ |
| 135 | Region of Peel | https://www.peelregion.ca/ |
| 136 | Region of Waterloo | https://www.regionofwaterloo.ca/ |
| 137 | Saskatchewan Early Childhood Association | http://seca-sk.ca/ |
| 138 | Scope BC | https://divisionsbc.ca/east-kootenay/our-impact/our-partners/scope-bc |
| 139 | Simcoe Muskoka District Health Unit | https://www.simcoemuskokahealth.org/ |
| 140 | Southwest Physical Activity Promoters Network | https://www.healthunit.com/physical-activity-early-years |
| 141 | Southwestern Public Health - Oxford-Elgin - St. Thomas | https://www.swpublichealth.ca/en/ |
| 142 | Speech Services Niagara | http://www.speechservicesniagara.ca/ |
| 143 | Success by 6 BC | https://www.successby6bc.ca/ |
| 144 | Success by 6 Edmonton | http://www.successby6edmonton.info/ |
| 145 | Timiskaming Health Unit | http://www.timiskaminghu.com/ |
| 146 | TOPP Kids | http://www.toppkids.com/ |
| 147 | Toronto Public Health | https://www.toronto.ca/ |
| 148 | Tri-Cities Kids Matter | https://www.tricitieskidsmatter.ca/ |
| 149 | Unplug and Play Week (Interior Savings) | http://unplugandplayweek.com/ |
| 150 | Vancouver Coastal Health | http://www.vch.ca/ |
| 151 | Wee R Kids | https://www.weerkids.net/ |
| 152 | West Coast Child Care Resource Centre | https://www.wstcoast.org/ |
| 153 | Wholesome Kids Catering | https://wholesomekids.ca/ |
| 154 | Windsor - Essex County Health Unit | https://www.wechu.org/ |
| 155 | Wymbin | https://www.wymbin.com/early-learning |
| 156 | YMCA of Greater Toronto | https://ymcagta.org/ |
| 157 | YMCA of Kamloops Community | https://www.kamloopsy.org/ |
| 158 | YMCA of Greater Vancouver | https://www.gv.ymca.ca/ |
| 159 | YMCA of Guelph | https://www.guelphy.org/ |
| 160 | YMCAs across Southwestern Ontario | https://www.ymcaswo.ca/ |
| 161 | York Region Community and Health Services Public Health | https://york.ca/ |
| 162 | Yukon Health and Social Services | https://hss.yukon.ca/ |
